# Biomass Production and Carbon Stocks in Poplar-Crop Agroforestry Chronosequence in Subtropical Central China

**DOI:** 10.3390/plants12132451

**Published:** 2023-06-26

**Authors:** Zhong Wang, Wende Yan, Yuanying Peng, Meng Wan, Taimoor Hassan Farooq, Wei Fan, Junjie Lei, Chenglin Yuan, Wancai Wang, Yaqin Qi, Xiaoyong Chen

**Affiliations:** 1 College of Life Science and Technology, Central South University of Forestry and Technology, Changsha 410004, Chinayuanchenglin0424@gmail.com (C.Y.);; 2 National Engineering Laboratory for Applied Technology in Forestry and Ecology in South China, Central South University of Forestry and Technology, Changsha 410004, China; 3 College of Arts and Sciences, Lewis University, Romeoville, IL 60446, USA; pengyu@lewisu.edu; 4 Henan Forestry Research Institute, Zhengzhou 450008, China; 5 Bangor College China, a Joint Unit of Bangor University and Central South University of Forestry and Technology, Changsha 410004, China; 6 Institute of Soil and Water Conservation, Northwest A&F University, Xianyang 712100, China; 7 State Key Laboratory of Remote Sensing Science, Aerospace Information Research Institute, Chinese Academy of Sciences, Beijing 100101, China; qiyq@aircas.ac.cn; 8 College of Arts and Sciences, Governors State University, University Park, IL 60484, USA

**Keywords:** productivity, carbon storage, sustainability, land use, multi-aged stands

## Abstract

Agroforest systems have been widely recognized as an integrated approach to sustainable land use for addressing the climate change problem because of their greater potential to sequester atmospheric CO_2_ with multiple economic and ecological benefits. However, the nature and extent of the effects of an age-sequence of agroforestry systems on carbon (C) storage remain largely unknown. To reveal the influence of different aged poplar-crop systems on C stocks, we investigated the variation in biomass and C storage under four aged poplar-crop agroforest systems (3-, 9-, 13-, and 17-year-old) in the Henan province of China. The results showed that stand biomass increased with forest age, ranging from 26.9 to 121.6 t/ha in the corresponding four aged poplar-crop systems. The poplar tree biomass accounted for >80% of the total stand biomass in these poplar-crop agroforestry systems, except in the 3-year-old agroforestry system. The average stand productivity peaked in a 9-year-old poplar-crop system (11.8 t/ha/yr), the next was in 13- and 17-year-old agroforestry systems, and the minimum was found in 3-year-old poplar-crop stands (4.8 t/ha/yr). The total C stocks increased, with aging poplar-crop systems ranging from 99.7 to 189.2 t/ha in the studied agroforestry systems. The proportion of C stocks accounted for about 6, 25, and 69% of the total C stocks in the crop, poplar tree, and soil components in all studied agroforestry ecosystems, respectively. Our results suggested that the poplar-crop system, especially in productive and mature stages, is quite an effective agroforestry model to increase the study site’s biomass production and C stocks. This study highlighted the importance of agroforestry systems in C storage. It recommended the poplar-crop agroforest ecosystems as a viable option for sustainable production and C mitigation in the central region of China.

## 1. Introduction

As atmospheric CO_2_ concentrations continue to rise due to human activity, we must seek viable management strategies to promote and enhance carbon (C) capture in terrestrial ecosystems to mitigate CO_2_-induced climate change [1,2,3]. Afforestation and reforestation have been proposed as favorable options for removing atmospheric CO_2_ through plant photosynthesis and storing it in tree bodies and soils [4,5,6,7]. Among the practical afforestation strategies, agroforestry is an important C storage strategy because it can provide both climate change mitigation and food recourses [8,9,10,11].

Agroforestry is the combination of trees and crops, which plays an increasing role in mitigating the harmful effects of global change and soil degradation [12,13,14]. Promoting agroforestry is one option to solve problems related to land use and CO_2_-induced global warming [15,16]. Agroforestry systems, directly and indirectly, affect C storage because of their greater potential to sequester atmospheric CO_2_ and other ecosystem services, including food security and soil conservation [17,18]. Agroforestry exploits the ecological and economic interactions of the different components [15,19]. In an agroforestry ecosystem, the trees have various functions, including shading crops to reduce evapotranspiration, erosion control, and nutrient cycling [20,21]. Depending on the species, the shade tree can be regularly pruned for soil improvement or left to produce firewood or timber [22,23].

Additionally, agroforestry is one of the best ways to improve environmental and socioeconomic sustainability because it offers a mix of market and non-market commodities and services such as food, fuel, wood products, water and air quality improvement, soil conservation, and nutrient enrichment, biodiversity conservation, and scenic beauty [24,25,26,27,28]. The historical evidence shows that agroforestry has been widely practiced through the ages to achieve agricultural sustainability and slow the negative effects of agriculture, such as soil degradation and desertification [22]. Several studies have shown that agroforestry systems often improve the productivity of both forest and agriculture systems while providing opportunities to create C sinks [13,29,30,31]. The biomass productivity in agroforestry systems depends on several factors, including tree ages, species structures, and how the system is managed [22]. On a global scale, the complex mixtures of trees and crops are widely practiced in Latin America, Southeast Asia, and Equatorial Africa. They are among the most sustainable cropping systems in the tropics [16]. The average C storage by agroforestry practices is 9, 21, 50, and 63 Mg C ha^−1^ in semiarid, sub-humid, humid, and temperate regions [8]. For smallholder agroforestry systems in the tropics, potential C storage rates range from 1.5 to 3.5 Mg C ha^−1^ yr^−1^ [8]. On average, C benefits are greater in agroforestry systems in tropical climates when compared to agroforestry systems located in other climates, both in terms of soil (2.23 Mg C ha^−1^ yr^−1^) and above ground (4.85 Mg C ha^−1^ yr^−1^) [32].

Forests of various ages fulfill distinct roles in C removal from the atmosphere and its storage within the wood. Older forests have accumulated a greater amount of C compared to younger forests. However, young forests exhibit rapid growth, enabling them to extract a significantly higher amount of CO_2_ from the atmosphere each year than an older forest of the same size. By managing forests in a manner that prevents substantial emissions resulting from the loss of old trees and simultaneously promoting the rapid absorption of CO_2_ through the growth of young forests, both storage and environmental benefits can be achieved. Furthermore, managed forests yield wood products that store C long after the trees are harvested. These products offer an additional advantage when used as alternatives to more energy-intensive options that contribute to higher fossil fuel emissions. There are always different thoughts on higher absorptions of C by younger and older tree stands, paving the way for more age-varied plantation-based studies. 

The dominant feature of China’s subtropical forests is the preservation of a remarkable evergreen forest ecosystem, which encompasses approximately 25% of the country’s land area [33,34]. Poplar (*Populus tomentosa* Carrière) is an important fast-growing tree species in subtropical southern China, with short rotation and high industrial requirements, also renowned worldwide [35], which produces a large amount of biomass per unit area in different planting systems [25]. However, the proper age structure of poplars is critical for high-performance productivity in different regions [36], and for soil organic carbon and nitrogen pools under intensive management [37]. It is significant to know that the most important benefit of planting poplar is the great capacity of poplar to absorb CO_2_ and stock C, with a determining role in phytoremediation and C fixation. One hectare planted with poplar can absorb up to 25 tons of CO_2_ [38]. It has been calculated that this theoretically corresponds to an economic value of about 1000 USD per year [38]. 

Although poplar is currently used in some tree-based intercropping systems in many countries, the age structure pattern of poplar biomass components, and their allocation and crop biomass in the field, is still unknown. In particular, the quantity, distribution, and proportion of C stocks are still unclear in various components of agroforestry ecosystems along a chronosequence. This study estimated biomass production and C inputs from plant organs to soils in replicated stands across a chronosequence of poplar-crop agroforestry systems (poplar–wheat–peanut). The study’s objectives were as follows: (1) to quantify the total biomass and biomass allocation, including poplar and crop biomass, and (2) to explore the soil C storage in four different aged agroforestry systems. 

## 2. Results

**Figure 1:**
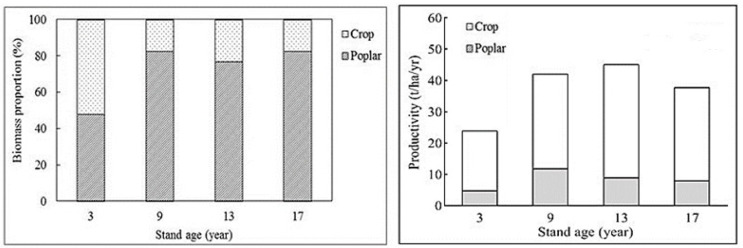
The proportion of poplar and crop biomass and variation in mean individual poplar-tree and poplar-crop stand productivity of different aged poplar-crop agroforestry systems at the study site.

The poplar tree accounted for about 80% of the total biomass of the poplar-crop agroforests in all exanimated stands, except the 3-year-old poplar stands where the poplar tree accounted for about 48% of the total biomass of the poplar-crop agroforestry (Figure 1). 

The tree productivity of poplar-crop stands increased from the 3-year-old stands (4.8 t/ha/yr) to the 9-year-old stands (about 11.8 t/ha/yr) and then reduced to the 13- and 17-year-old stands (about 8.5 t/ha/yr) (Figure 1). The highest total stand productivity occurred in the 13-year-old poplar-crop agroforests, while the lowest was found in the 3-year-old poplar-crop agroforests (Figure 1). 

**Figure 2:**
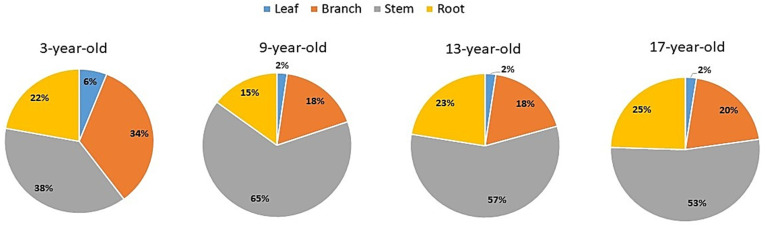
Percentage of biomass allocation in different tree organs of individual trees in different aged stands of poplar-crop agroforestry systems.

The mean total biomass of individual poplar trees ranged from 30.84 to 393.12 kg across four aged poplar-crop agroforests. The biomass of each tree organ also increased with forest age (Table 1). The percentage of different tree organs in the total tree biomass decreased in the order of stem > branches > roots > leaves throughout the four aged poplar-crop agroforests. In particular, the stem organ accounted for more than 50% of the total tree biomass, except in 3-year-old forests, where the percentage of the stem was about 38% of the total tree biomass. The percentage proportion of biomass allocation in different tree organs of individual trees is shown in Figure 2.

The biomass of wheat and peanut crops ranged from 14.05 to 27.15 with an order of 13- > 17- > 9- > 3-year-old. The total stand biomass of the poplar-crop agroforests ranged from 26.9 to 121.6 t/ha and increased as the age increased (Table 2). 

**Figure 3:**
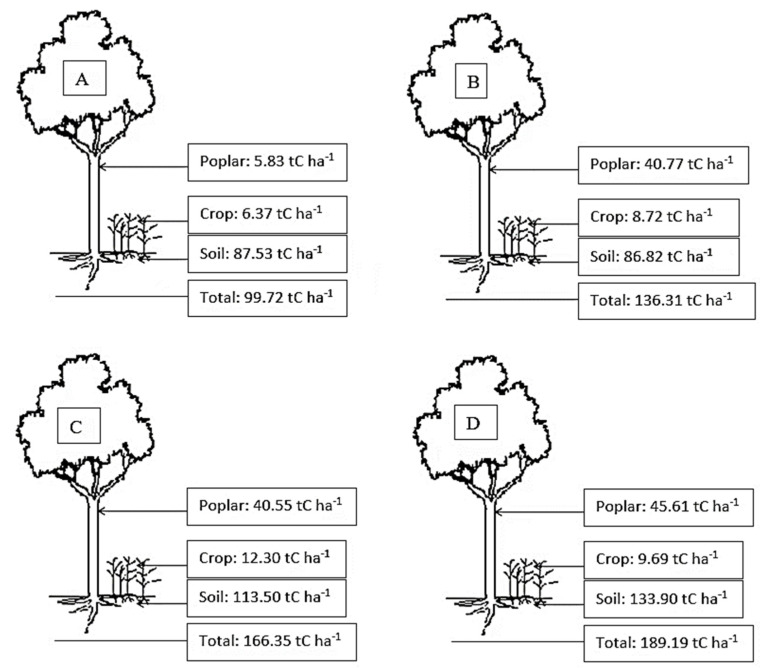
Distribution of carbon in different components of the poplar-crop agroforest ecosystems in the study site. (**A**): 3-year-old stands, (**B**): 9-year-old stands, (**C**): 13-year-old stands, and (**D**): 17-year-old stands.

**Figure 4:**
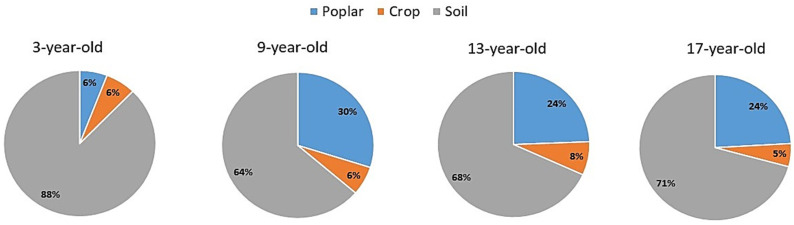
Percentage of carbon allocation in different layers of poplar-crop agroforest ecosystem in the study site. (**A**): 3-year-old stands, (**B**): 9-year-old stands, (**C**): 13-year-old stands, and (**D**): 17-year-old stands.

The total C storage in the four aged stands of poplar-crop agroforests increased with increasing stand age, ranging from 99.72 to 189.19 Mg C ha^−1^ (Figure 3). In poplar-crop agroforest ecosystems of different ages, poplar components accounted for about 25–30% of the total C pool, but in the 3-year-old stand, poplar components only accounted for about 6% of the total C pools in agroforestry ecosystems. Most of the C (about 65–70%) was stored in the soil in the studied poplar-crop agroforest ecosystems. The crop component only accounted for about 10% of C stocks in the poplar-crop agroforest ecosystem in the study area (Figure 3). The percentage proportion of C allocation in different layers of poplar-crop agroforest ecosystems is shown in Figure 4.

## 3. Discussion

Mature poplar stands (17 years old) had the highest stand biomass (121.64 t/ha). The total biomass of the poplar tree layer in four aged stands increased as the ages increased, and was significantly higher than in the other stands. This phenomenon should be attributed to poplar plantation growth and associated with wheat–peanut crop production in agroforestry systems. Poplar-investor cultivators seem to be a long-term investment compared to annual crops [39]. Our result was higher than that for the previous observation in poplar agroforestry systems in China; for example, the largest total C stock reached 16.7 t C ha^−1^ for the poplar with a wheat–corn combination, and 18.9 t C ha^−1^ with a wheat–soybean cropping system observed in northwestern Jiangsu, China [40]. Our findings are in line with the findings in poplar-based agroforestry systems in India [41]; they found that the total C storage was about 112.5, 101.8, 84.9, 77.3, and 38.8 t C ha^−1^ in five different plant spacing geometries, respectively. 

In this study, the greatest C storage for 17-year-old stands was 45.61 t/ha, followed by 13-year-old (40.55 t/ha), 9-year-old (40.77 t/ha), and 3-year-old (5.83 t/ha) intercropped poplar. According to other literature reviews, the C storage in 5-year-old poplar plantations ranged from 4.5 to 7.6 C ha^−1^ [40]. The average C storage by agroforestry practices has been estimated to be 9, 21, 50, and 63 Mg C ha^−1^ in semiarid, subhumid, humid, and temperate regions. The highest C storage of 6.5 mg ha^−1^ was observed in 8-year-old poplar-based agroforestry in the Mazandaran province, in the north of Iran [42]. While comparing the biomass production and C stock capacity for individual trees, the highest biomass production and C storage of individual poplar trees were found in 9-year-old stands (32.9 kg dry mass/tree/yr and 15.0 kg C/tree/yr), for which values were about 3.2, 1.2, and 1.4 times higher than those in 3-, 13-, and 17-year-old stands. At the poplar-crop community level, the pattern of biomass production for the four aged poplar agroforestry systems was 9-year-old stand > 3-year-old stand > 13-year-old stand > 17-year-old stand. 

As the stand biomass was related to the dry mass of individual trees and the tree number per unit of area, stand density might play a critical role in accumulating biomass production and C stocks in agroforestry systems [36,43]. The highest total biomass production and C storage in crops were found in 13-year-old stands, also because of the high stand density at 255 trees/ha in 17-year-old stands compared to the low stand density at 225 trees/ha in 13-year-old stands. The low density of the stand provided more space for crops to grow [44]. These results had a similar pattern to the results proposed by [43] and [45]; estimating the C fixation relies on site-specific factors such as stand density [43]. 

Individual trees grew larger in diameter with low densities because of a large planting space with more soil nutrients [44]; however, fewer trees per unit area at the lower densities do not increase total stand biomass production [46,47]. Numerous smaller trees can produce an equivalent amount of biomass compared to fewer larger trees planted in the same stands. This means that over the range of densities in stands, the establishment costs can be increased compared with planting fewer trees, without compromising productivity [44,46,47]. It should be noted that the biomass production and C storage approach do not factor in indirect emissions related to farm operations and cultivation inputs; therefore, the biomass approach provides estimates of the C storage potential rather than the complete C balance of agroforestry ecosystems [43].

Intercropped wheat and peanut were assumed to produce half of the average yield of crops produced as sole crops. Although there are no data to support the assumption, this assumption was justified by the fact that understory biomass production is affected by canopy closure in hybrid poplar systems [40,44]. Fang et al. [43] indicated that the reduction in wheat production varied from a minimum of 1.3% to a maximum of 14.8% depending on spacing and the growth rate of poplars at the 4-year-old stand age. In comparison, the increase in biomass productivity and light-use efficiency varied from 15.1 to 38% and from 18.8 to 43.8%, respectively, compared with crops alone. Similarly, in mature poplar plantations with a stand density of 255 trees ha^−1^, the percentage of crops intercropping with tree species was estimated to be 15–20% that of an open field in hay biomass production. Wall et al. [48] found that hay biomass was likely to decrease significantly with canopy closure. Still, they could not be more precise in predicting the yield due to a lack of information on hay yields in hybrid poplar–hay intercropping systems. 

## 4. Materials and Methods

### 4.1. Study Site

**Figure 5:**
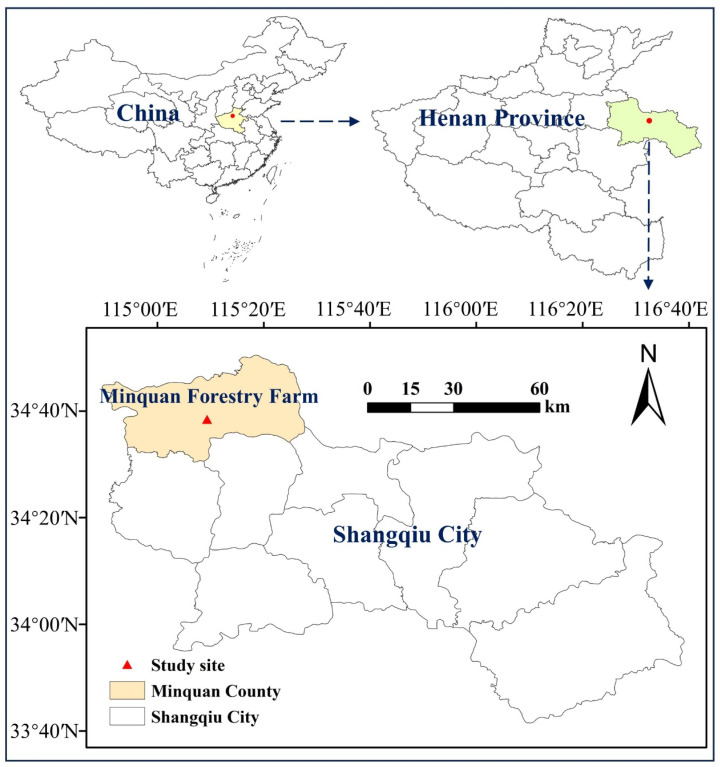
The study area is Minquan Forestry Farm, Minquan County, Henan Province, China.

The study was conducted in Minquan Forestry Farm, Minquan County, Henan province, China (34°31′–34°52′ N, 114°–115°28′ E) (Figure 5). The terrain features a relatively open topography, with low slopes at an elevation of approximately 61 m above sea level. The climate is typically a warm temperate continental monsoon with four distinct seasons: dry/windy spring, intensive/hot summer, fine/comfortable autumn and dry/cold winter with a mean annual temperature of 14.0 °C. The mean annual precipitation is approximately 679 mm, with about 75% falling during June and August. The soils in the study site were a deep and fine sandy earth type, derived from fluvial transport and deposition from the Yellow River. The texture was sandy loam. The soils had low fertility, with an organic matter content of 0.27–0.36%, pH value of 7.0–9.0, total nitrogen content of 0.01 to 0.03%, available P content of 1.2 to 3.7 mg kg^−1^, available K contents of 32.4 to 57.6 mg kg^−1^, and organic C content of 0.16 to 2.1%. The water table fluctuated between 1.0 and 4.0 m from the soil’s surface.

The study area is floristically representative of poplar plantation forests in central China. Because of the characteristics of fast growth, windy protection, and soil conservation, poplar plantations have been largely developed and built up as the dominant tree species in agroforestry systems in the central regions of China since the 1970s. Other dominant native tree species include *Robinia pseudoacacia*, *Catalpahungei* C. A. Mey, *Koelreuteria paniculata* Laxm, and *Ailanthus altissina* Swingle. Dominant shrub species include *Buxus sinica* Cheng, *Morus alba* Linn, *Ligustrun lucidum* Ait, and *Cynanchum thesioides* K. The dominant herbaceous plants include *Eleusine indica* Gaertn, *Digitaria sanguinalis* L. *Humulus scandens* Merr, and *Rubia cordifolia* L.

### 4.2. Experimental Design

The experiment was a completely randomized design (CRD). The four aged poplar plantations with 3-, 9-, 13-, and 17-year-olds were chosen for this study. These selected aged categories represented four distinct phases of growth development in poplar agroforestry systems: the planting and the early development stage (3-year), the productive stage (9-year), the near-mature stage (13-year), and the mature phase (17-year). Based on the stand density and the planting spacing, 22 plots were established in the four aged plantation groups with 5, 5, 6, and 6 plots in 3-, 9-, 13-, and 17-year-old plantations, respectively. The area of the plot was 16 × 54 m^2^, 20 × 33 m^2^, 64 × 33 m^2^, and 20 × 33 m^2^ in 3-, 9-, 13-, and 17-year-old plantations stands, respectively. The sampling locations were randomly selected with a minimum of 4 m distances between each other. All these poplar plantations were established using the same method. 

For crops, each crop had five quadrates (1 × 1 m) to set up in each of the stand sampling plots to measure the crop’s biomass. The total ten quadrats (wheat and peanuts) were randomly placed in each poplar intercropping plot based on the distance of the tree rows. The characteristics of the four aged stands of the poplar forests are listed in Table 3.

### 4.3. Measurements of Biomass

#### 4.3.1. Measurements of Biomass in Poplar Stands and C Storage

The study measured biomass in 3-, 9-, 13-, and 17-year-old poplar stands. Poplar trees were partitioned into stem, bark, branch, leaf, and root components. Biomass was estimated using the ‘six sample tree’ method. All trees in the experimental plots were numbered and divided into five categories: dominant, sub-dominant, medium, suppressed, and nearly dead trees based on the size dimension of DBH (diameter at the breast height, 1.3 m above the ground) and the height of the individual tree and the growth status. The DBH and height of all trees were recorded in each plot. 

Five trees separately represented the five categories (the average dimension of DBH and tree height of five categories) from all individual trees in each plot, plus one extra tree, which represented the overall average dimension of all individual trees in the plot, and together these were chosen as the sampling trees for biomass measurements. To protect the structure of the experimental plot, the six sampling trees were selected and logged outside, but close to the experimental plot, based on the growth status and the tree dimension of the average size marked in the experimental plot. The six sampled trees were cut down just above the root collar. After felling the sampled tree, all branches with leaves were removed based on a 2 m vertical layer interval from the bottom to the top of the crown. The fresh weights of the branches at each layer were weighed in the field. Two branches representing the average dimension of all branches at each layer were selected as subsamples for dry weight determination. The leaves in the subsamples of branches were stripped to determine the ratio of branch to leaf, and the fresh-to-oven-dried weight ratio for each layer of a sampled tree crown.

Each stem was cut into 2 m long sections. The fresh weight of each section was determined directly in the field. A stem disc (about 2 cm wide) from each section was taken as a subsample, and the bark was stripped from the stem in each section to determine the stem-to-bark ratio and fresh-to-oven-dried weight ratio for each section of the sampled tree stem. Each sampled tree’s roots were carefully excavated from different soil layers (0–20, 20–50, and 50–100 cm depth) to a depth of 1.0 m. Roots were separated manually from the soil and were gently rinsed with water. All layers and classes of the fresh-weight root biomass were weighed in the field. Root subsample components from each layer and each class were taken into the lab to measure the fresh-to-oven-dried weight ratios at 70 °C to obtain a constant weight. The branch-to-leaf ratio, stem-to-bark ratio, and fresh-to-oven-dried weight ratios were used to convert fresh and dry weights for each sampled tree component. The biomass and the components of biomass for each tree were calculated.

The equation for the estimation of biomass as a function of individual poplar tree diameter (DBH in cm) and tree height (H in meter) was

W = a × (D^2^ × H)^b^

where W is biomass, a presented intercept coefficient, B is the scaling exponent, and R^2^ is the coefficient of correlation relationship (Table 4).

#### 4.3.2. Measurements of Crop Biomass and C Storage

The full harvesting method was applied to measure the total biomass of wheat and peanut crops in all quadrates. All biomasses were weighed fresh and then were taken in the lab to measure the dry weights to determine the fresh-to-oven-dried weight ratio of crops. The total crop biomass was measured by cutting aboveground, excavating belowground roots, and then drying and weighing all the plant material within the quadrates. Root excavation extended downwards to 20–30 cm until no roots were visible. The fresh weights of all components for each quadrate were measured in the field, and sub-samples for each component were collected for moisture and C analysis. The crop sampling biomass was measured in all quadrates, and then the total crop biomass was calculated based on the whole area of the plot.

The C content of the tree and crop biomass components was quantified for all plots. The C content of the plants was measured using a CNH autoanalyzer. The C stock in the biomass in each stand is termed as the ‘C pool in plants’, including all components of poplar trees and crops.

### 4.4. Statistical Analyses

Analysis of variance (ANOVA) was used to assess the effects of four ages of poplar statistical stands intercropping with crops on plant biomass, crop biomass, and C content. The above- and belowground biomass data were log-transformed to satisfy ANOVA’s normality and homoscedasticity assumptions. The means of aged stands and their combinations were compared by a Tukey–Kramer test. Statistical analyses were conducted using a SAS statistical package (SAS Institute, Inc., Cary, NC, USA, 1999–2001). 

## 5. Conclusions

Agroforestry systems have the potential to sequester C with the adequate management of trees in cultivated lands and pastures. Intercropping systems could capture and store a significant fraction of the atmospheric C in plant biomass and soils. This study proves that yields measured after 9 years of poplar growth have high biomass and C storage. Our research results indicate that poplar plantations at productive and mature stages of intercropping with crops are quite an effective agroforestry model, providing a high efficiency of biomass production and C stocks as well as ecological benefits in southern China. Our research provides scientific insight into the efficiency of C storage in the long-term growth of poplar trees and soil fertility. 

## Figures and Tables

**Table 1:** Biomass of different tree components of individual trees and their proportion in different aged stands of poplar-crop agroforestry systems (standard deviation).

Stand Age (yr)	Biomass of Individual Tree (kg)				Total
	Leaf	Branch	Stem	Root	
3	1.85	10.42	11.75	6.82	30.84 (5.32)
9	6.63	51.92	193.73	43.88	296.16 (16.35)
13	8.11	63.84	199.47	78.25	349.67 (25.45)
17	9.36	79.96	207.36	96.44	393.12 (19.36)

**Table 2:** Biomass of poplar tree, wheat, and peanut in different aged poplar-crop agroforestry systems in the study site (standard deviation).

Stand Age (yr)	Poplar Biomass (t/ha)					Crop Biomass (t/ha)			Overall Total Biomass (t/ha)
	Stem	Branch	Leaf	Root	Total	Wheat	Peanut	Total	
3	4.88	4.32	0.77	2.83	12.80 (1.3)	5.28	8.77	14.05 (0.9)	26.85 (2.4)
9	58.70	15.73	2.01	13.30	89.74 (8.3)	6.95	12.30	19.25 (1.4)	108.99 (10.2)
13	50.86	16.28	2.07	19.95	89.17 (9.7)	10.25	16.90	27.15 (2.5)	116.32 (10.8)
17	52.88	20.39	2.39	24.59	100.25 (11.1)	7.55	13.84	21.39 (2.1)	121.64 (12.2)

**Table 3:** Characteristics of the selected four aged stands of poplar forests in the study site (± standard deviation).

Stand Age (yr)	Stand Density (Tree/ha)	DBH (cm)	Height (m)	Crown Width (m^2^)	Planting Spacing (m)
3	415 ± 26	9.0 ± 2.7	7.4 ± 2.8	2.9 × 3.1	3 × 8
9	303 ± 21	24.0 ± 3.9	21.9 ± 3.4	2.8 × 3.6	3 × 10
13	225 ± 27	40.0 ± 3.1	26.5 ± 3.9	3.7 × 7.3	8 × 10
17	255 ± 24	42.6 ± 4.8	27.2 ± 3.5	4.3 × 6.3	8 × 10

**Table 4:** The allometric equation for estimation of biomass as a function of tree diameter at breast height (D, cm) and tree height (H, m) was W = a × (D^2^ × H)^b^, where W: biomass; a: intercept coefficient; b: scaling exponent; R^2^: coefficient of determination.

Tree Organs	a	b	R^2^
Leaf	0.0010	1.1902	0.9001
Branch	0.0146	0.9174	0.9282
Stem	0.0810	1.0658	0.9982
Root	0.0199	0.8409	0.9485

## Data Availability

The corresponding authors will provide data upon formal request.
